# Which New Health Technologies Do We Need to Achieve an End to HIV/AIDS?

**DOI:** 10.1371/journal.pbio.1002372

**Published:** 2016-03-02

**Authors:** Glenda E. Gray, Fatima Laher, Tanya Doherty, Salim Abdool Karim, Scott Hammer, John Mascola, Chris Beyrer, Larry Corey

**Affiliations:** 1 South African Medical Research Council, Cape Town, South Africa; 2 Perinatal HIV Research Unit, Faculty of Health Sciences, University of the Witwatersrand, Johannesburg, South Africa; 3 School of Public Health, University of Western Cape, Bellville, South Africa; 4 School of Public Health, University of the Witwatersrand, Johannesburg, South Africa; 5 Centre for the AIDS Programme of Research in South Africa, University of KwaZulu-Natal, Durban, South Africa; 6 Mailman School of Public Health, Columbia University, New York City, New York, United States of America; 7 Columbia University Medical Center, New York City, New York, United States of America; 8 Vaccine Research Center, National Institute of Allergy and Infectious Diseases, National Institutes of Health. Bethesda, Maryland, United States of America; 9 Johns Hopkins Bloomberg School of Public Health, Baltimore, Maryland, United States of America; 10 The International AIDS Society, Geneva, Switzerland; 11 HIV Vaccine Trials Network, Vaccine and Infectious Disease Division, Fred Hutchinson Cancer Research Center, Seattle, Washington

## Abstract

In the last 15 years, antiretroviral therapy (ART) has been the most globally impactful life-saving development of medical research. Antiretrovirals (ARVs) are used with great success for both the treatment and prevention of HIV infection. Despite these remarkable advances, this epidemic grows relentlessly worldwide. Over 2.1 million new infections occur each year, two-thirds in women and 240,000 in children. The widespread elimination of HIV will require the development of new, more potent prevention tools. Such efforts are imperative on a global scale. However, it must also be recognised that true containment of the epidemic requires the development and widespread implementation of a scientific advancement that has eluded us to date—a highly effective vaccine. Striving for such medical advances is what is required to achieve the end of AIDS.

## Abstract 2

This essay argues that while the tools to control the HIV epidemic are already at our disposal, to end AIDS we need innovative strategies for HIV vaccine design, microbicide platforms, and an HIV cure.

In the last 15 years, antiretroviral therapy (ART) has been the most globally impactful life-saving development of medical research. Antiretrovirals (ARVs) are used with great success for both the treatment and prevention of HIV infection. In the United States, the widespread implementation of combination ARVs led to the virtual eradication of mother-to-child transmission of HIV from 1,650 cases in 1991 to 110 cases in 2011, and a turnaround in AIDS deaths from an almost 100% five-year mortality rate to a five-year survival rate of 91% in HIV-infected adults [1]. Currently, the estimated average lifespan of an HIV-infected adult in the developed world is well over 40 years post-diagnosis. Survival rates in the developing world, although lower, are improving: in sub-Saharan Africa, AIDS deaths fell by 39% between 2005 and 2013, and the biggest decline, 51%, was seen in South Africa [2].

Furthermore, the association between ART, viremia, and transmission has led to the concept of “test and treat,” with the hope of reducing community viral load by testing early and initiating treatment as soon as a diagnosis of HIV is made [3]. Indeed, selected regions of the world have begun to actualize the public health value of ARVs, from gains in life expectancy to impact on onward transmission, with a potential 1% decline in new infections for every 10% increase in treatment coverage [2]. In September 2015, WHO released new guidelines removing all limitations on eligibility for ART among people living with HIV and recommending pre-exposure prophylaxis (PrEP) to population groups at significant HIV risk, paving the way for a global onslaught on HIV [4].

Despite these remarkable advances, this epidemic grows relentlessly worldwide. Over 2.1 million new infections occur each year, two-thirds in women and 240,000 in children [2]. In heavily affected countries, HIV infection rates have only stabilized at best: the annualized acquisition rates in persons in their first decade of sexual activity average 3%–5% yearly in southern Africa [5–7]. These figures are hardly compatible with the international health community’s stated goal of an “AIDS-free generation” [8,9]. In highly resourced settings, microepidemics of HIV still occur, particularly among gays, bisexuals, and men who have sex with men (MSM) [10]. HIV epidemics are expanding in two geographic regions in 2015—the Middle East/North Africa and Eastern Europe/Central Asia—largely due to challenges in implementing evidence-based HIV policies and programmes [2]. Even for the past decade in the US, almost 50,000 new cases recorded annually, two-thirds among MSM, has been a stable figure for years and shows no evidence of declining [1].

While treatment scale-up, medical male circumcision [11], and the implementation of strategies to prevent mother-to-child transmission [12] have received global traction, systemic or topical ARV-based biomedical advances to prevent sexual acquisition of HIV have, as yet, made limited impressions on a population basis, despite their reported efficacy. Factors such as their adherence requirements, cost, potential for drug resistance, and long-term feasibility have restricted the appetite for implementation, even though these approaches may reduce HIV incidence in select populations.

Already, several trials have shown that daily oral administration of the ARV tenofovir disoproxil fumarate (TDF), taken singly or in combination with emtricitabine, as PrEP by HIV-uninfected individuals, reduces HIV acquisition among serodiscordant couples (where one partner is HIV-positive and the other is HIV-negative) [13], MSM [14], at-risk men and women [15], and people who inject drugs [16,17] by between 44% and 75%. Long-acting injectable antiretroviral agents such as rilpivirine and cabotegravir, administered every two and three months, respectively, are also being developed for PrEP. All of these PrEP approaches are dependent on repeated HIV testing and adherence to drug regimens, which may challenge effectiveness in some populations and contexts.

The widespread elimination of HIV will require the development of new, more potent prevention tools. Because HIV acquisition occurs subclinically, the elimination of HIV on a population basis will require a highly effective vaccine. Alternatively, if vaccine development is delayed, supplementary strategies may include long-acting pre-exposure antiretroviral cocktails and/or the administration of neutralizing antibodies through long-lasting parenteral preparations or the development of a “genetic immunization” delivery system, as well as scaling up delivery of highly effective regimens to eliminate mother-to-child HIV transmission (Fig 1).

**Fig 1:**
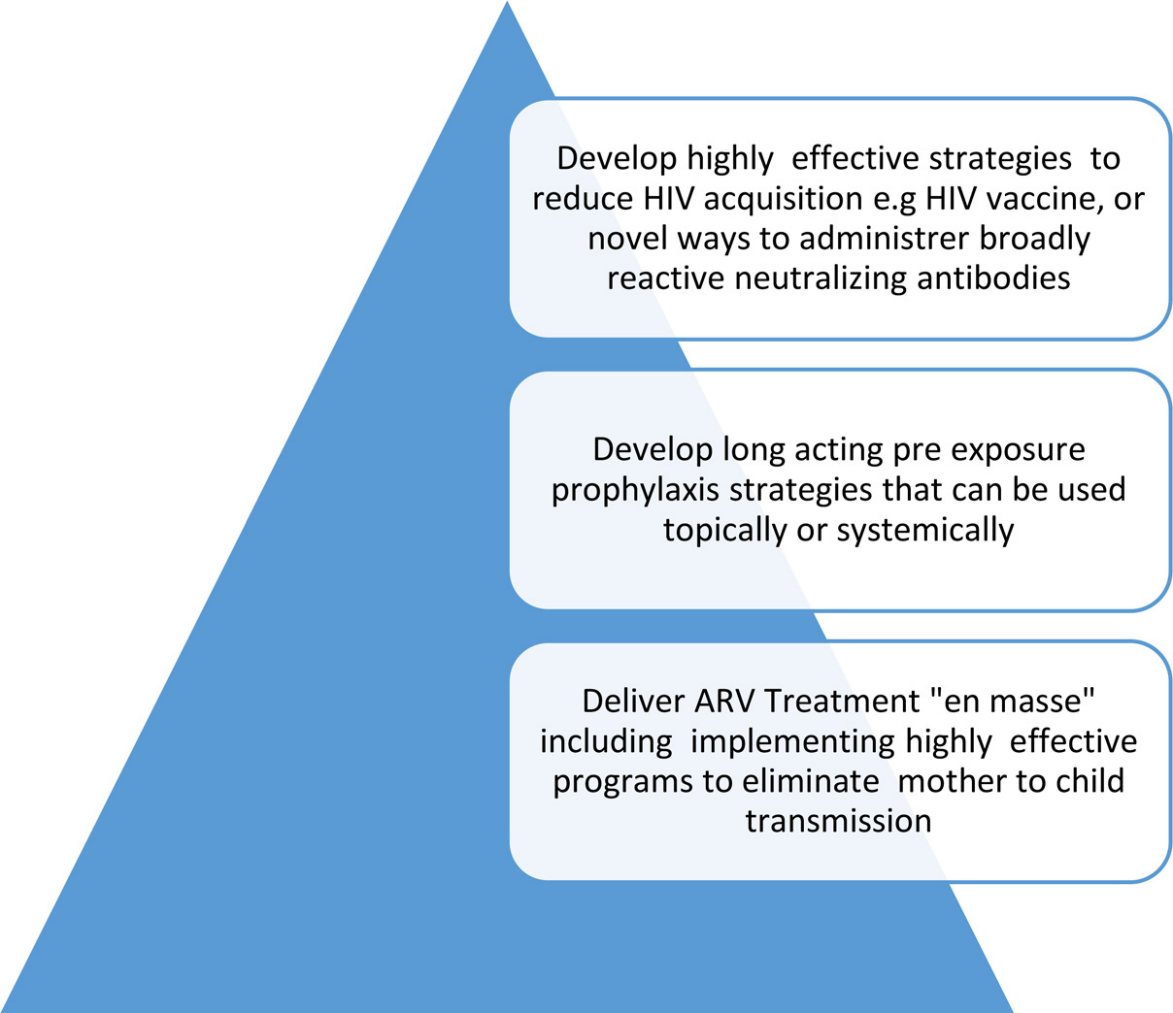
Medical interventions required to end the epidemic of HIV. Image credit: Glenda Gray.

## The Quest for an HIV Vaccine

HIV vaccine development has been challenging. The failure of five vaccine efficacy trials [17–21] and the modest efficacy of one [18–22] has limited the pharmaceutical industry’s role in HIV vaccine science. Current funding for HIV vaccine development is almost solely through government or philanthropic foundations [23]. The various approaches tested in late-stage clinical development, employing either viral vectors alone [17,18], in combination with DNA plasmids [21] or glycoprotein 120 [20], or glycoprotein 120 alone [19,20], have either been futile or have increased susceptibility to HIV acquisition in certain subgroups of vaccine recipients [17,18].

However, a milestone in the field was reached in late 2009, when the RV144 trial [24] of a recombinant canary pox prime and protein boost demonstrated modest efficacy with waning durability from 60% at 12 months to 31% at 36 months against sexually acquired HIV in Thailand. Although this vaccine regimen did not induce neutralizing antibodies, an immune correlates analysis indicated that it induced antibodies to the scaffolded V1V2 region of Env and that this response was associated with a reduced risk of infection [25,26]. Vaccine recipients who did not develop antibodies to the V1V2 scaffold demonstrated no vaccine efficacy, while those who developed antibodies to the V1V2 scaffold had an overall vaccine efficacy of 69% [27]. Additional analyses demonstrate that these V1V2 antibodies could mediate antibody-dependent cellular cytotoxicity (ADCC) and that the vaccine regimen induced immune pressure on viral sequences in the V1V2 region, providing increased support that this region is an important point of “attack” for nonneutralizing antibodies [28]. Other immune responses associated with vaccine efficacy in RV144 included polyfunctional CD4+ T cell responses as well as antibodies to envelope proteins of the IgG3 subclass [26,29]. These data, which suggest that nonneutralizing antibodies, especially to the V1V2 region of the HIV envelope, are associated with vaccine-induced protection, are provocative and require corroboration, as this V1V2 region had heretofore been largely ignored as a critical component of the human response to HIV.

Studies are underway to determine whether enhancing such responses by developing related vaccine prototypes with better T-cell priming as well as enhanced antibody responses will reproduce and enhance the vaccine efficacy seen in RV144. The Pox Protein Public Private Partnership (P5) has designed an HIV vaccine program to be executed in the clade-C-predominant areas of southern Africa. The program will initially evaluate the role of alternate adjuvants coupled with a bivalent clade C protein in combination with a clade C optimised ALVAC vector as a means of developing a more potent and durable antibody response. More recently, with initial funding from the US National Institutes of Health (NIH), a group of collaborators at the Ragon Institute, the US Military HIV Research Program (USMHRP), the International AIDS Vaccine Initiative (IAVI), and, more recently, Jansen Pharmaceuticals have initiated development of a vaccine regimen that consists of a replication defective Adenovirus type 26 and Modified Vaccinia Ankara (MVA) vaccine in combination with a gp 140 protein boost that incorporates a series of synthetic Mosaic genes designed to make immune responses to all the major clades of HIV. Nonneutralizing antibodies appear to be the major correlate of immune protection in nonhuman primate (NHP) challenge models. These vaccines are now entering expanded human clinical trials.

Other approaches to develop an effective HIV vaccine are also underway and include novel vector strategies as well as structural biology-based approaches. A recent study demonstrated that a live replicating cytomegalovirus (CMV) vaccine encoding several simian immunodeficiency virus (SIV) proteins, administered prior to SIV challenge, can lead to complete viral suppression and elimination of detectable SIV infection in about half of the rhesus macaques studied, implying that some lentiviral infections may be susceptible to clearance via effector memory T cell-mediated mechanisms [30]. This study provides new insights into the role of effector memory CD8 T-cells in control and elimination of SIV infection and, more generally, how to elicit long-term tissue resident immune responses at the mucosal sites where HIV acquisition occurs. Whether similar immune responses are translatable to humans and can lead to continued clinical development of this live attenuated vaccine approach remains to be determined. In addition, further evaluation of the immune responses of the macaques that did not clear their SIV infection is imperative to further the understanding of the correlates of sustained SIV infection post CMV vaccination.

A worldwide effort to isolate broadly neutralizing antibodies to HIV and to understand the structural basis for their neutralization as well as the immunological basis for their development [31–33] has brought insights into the design of new candidate vaccines as well as antibody-targeted approaches.

A major impediment to advancing HIV vaccine development has been the painstakingly slow pace of conducting vaccine efficacy trials, largely due to the difficulty in producing good manufacturing practice (GMP) quantities of novel HIV envelope proteins or recombinant vectors with novel inserts. After RV144, it took five years to manufacture the next generation of recombinant pox/recombinant gp120 proteins. The effective manufacturing of novel, often complex, immunogens has occurred almost exclusively in an industry where much of the “lore” of consistently manufacturing reproducible amounts of biologics is collectively learned over time. Engaging industry to actively embrace such high-risk ventures has been difficult, but it is a necessary hurdle for the HIV vaccine field to solve. Developing such resolve and expertise is difficult in a field in which scientific pluralism is valued and required.

Despite the challenge of developing an HIV vaccine, mathematical modelling has verified the profound impact a vaccine with variable coverage—even low-efficacy vaccines in combination with other interventions—would have at a population level [34,35].

## Neutralizing Antibodies for Prevention of HIV Acquisition

Numerous studies over the past 15 years have demonstrated the ability of HIV-1 neutralizing antibodies to prevent the acquisition of simian–human immunodeficiency virus (SHIV) infection in NHPs [36–39]. Technologies to manufacture these antibodies in sufficient quantities are now available, enabling test-of-concept trials exploring whether the infusion of one or more antibodies could persistently reduce viremia in HIV-infected persons.

Perhaps more importantly, human clinical trials are being convened, exploring whether infusion of monoclonal antibodies could prevent HIV acquisition. If clinical trial proof was obtained that neutralizing antibodies could markedly reduce HIV acquisition, it would be a conceptual breakthrough in the HIV prevention landscape and would validate the need to develop a neutralizing antibody vaccine. Additionally, these antibodies could either be administered via long-term infusion or provided in a continuous concentration through “genetic immunization” of the antibody using a persistent vector such as adeno-associated virus vectors or via the insertion of the HIV antibody gene in plasmablasts [40]. Applying the knowledge gleaned from cancer, autoimmune, and inflammatory diseases to increase the potency of antibodies by improving effector function of antibodies, such as enhancing ADCC and increasing serum half-life of IgG through Fc engineering [41–43], may improve the utility of this intervention for HIV treatment and prevention.

## Microbicides as an Innovation for HIV Prevention

Unlike the impressive breakthroughs with treatment, the quest to find potent biomedical prevention modalities for women’s routine use remains elusive. In sub-Saharan Africa, more than two-thirds of infections occur among women aged between 15 and 24 years. Young women typically acquire HIV infection 5–7 years earlier than their male counterparts [44,45]. HIV infections among women, especially young women, can be as much as 8-fold higher than in men of the same age [46] and is an important driver of the epidemic in Africa [47]. Candidate microbicides are being developed in an array of formulations, including gels, vaginal rings, rapidly dissolving vaginal tablets, films, and gel-filled compartments in diaphragms.

In 2010, the CAPRISA 004 trial, evaluating a coitally-dependent tenofovir gel regimen, first proved that ARVs can prevent sexual acquisition of HIV in women [48], followed by a study with MSM demonstrating the effectiveness of oral ARV chemoprophylaxis [14]. Adherence markedly affects the effectiveness of all PrEP regimens, and suboptimal adherence remains a large challenge for microbicide development [48,49], which was seen again in FACTS 001, a recent phase III confirmation study of coitally applied tenofovir gel [50]. To overcome these problems, long-acting, slow-release products are under development, including dapivirine or tenofovir in a vaginal ring [51,52]. In the future, multipurpose prevention technologies (e.g., for contraception and HIV prevention) and combination antiretroviral agent microbicides are in the works [53]. Rectal microbicide formulations for men and women at risk of HIV acquisition through anal intercourse are also under investigation [54].

## ARV Treatment: New Drugs and Formulations: Do We Have What It Takes?

The continued development of safer, better priced, and more convenient ARVs has resulted in the ongoing improvement of health for HIV-infected persons on both an individual and population basis. In the developed world, over a million deaths were averted by ARV treatments in the last decade.

Still, there were 1.5 million AIDS-related deaths during 2013, many of them potentially preventable, as 60% of HIV-infected people and 76% of HIV-infected children still need to be placed on treatment [2]. The most formidable barrier to the ubiquitous use of treatment is access. Communities and health systems experience enormous infrastructural and human resource shortcomings to early testing and treatment, drug supply, and retention in care. However, important innovations in drugs, treatment strategies, and monitoring are emerging that can solidify the expansion and resilience of treatment programs.

A drug development priority in a population-based scale-up of ART is a first-line single tablet that requires minimal laboratory monitoring and possesses minimal side effects with low rates of HIV resistance. Two recent advances on the classic TDF/emtricitabine/efavirenz first-line single tablet regimen promise to move us closer to this. First has been the coformulation of once-daily integrase inhibitors such as elvitegravir/cobicistat or dolutegravir. The latest US guidelines adopt the use of an integrase inhibitor with two nucleoside reverse transcriptase inhibitors as first-line therapy. In clinical trials, the single-pill TDF/emtricitabine/elvitegravir/cobicistat coformulation showed better safety profiles compared to its efavirenz-based peer [55]. Dolutegravir with a Nucleoside/Nucleotide Reverse Transcriptase Inhibitor (NRTI) backbone demonstrated superior ability to suppress viral load and better tolerability compared to efavirenz [56]. The second improvement has been the development of TDF’s pro-drug, tenofovir alafenamide fumarate (TAF), which has also been coformulated in a single daily pill with emtricitabine/elvitegravir/cobicistat. The latter coformulation showed virologic responses similar to its TDF equivalent but had better renal and bone profiles. In the developing world, future scale-up could be eased by prioritizing in-country licensure and cost reduction of such new drugs.

Even with maximum improvements on a single daily pill, ultimate scale-up must consider critical issues affecting retention in care, such as the lack of money for transport [57] and the challenges of getting time off work for patients to attend a clinic and collect medication. Drug delivery approaches that would guarantee longer-term adherence, such as drug implants, are being studied. Furthermore, the landscape of health staffing may need to transform, as the necessity of increasing personnel to handle increased patient volumes may require shifting tasks even further from nurses to trained counsellors to assist with monitoring and retention in care (Table 1).

**Table 1:** Innovations for scaling up care and keeping people on treatment.

Intervention	Innovation
**Diagnostics**	Non-blood-based diagnostics that are available “over the counter”
**Antiretroviral Treatment**	• Coformulations of once-daily pills with minimal side effects and high genetic barrier for resistance
	• Long-acting antiretrovirals minimising need for regular interface with health care providers
**Virological and Immunological Monitoring**	Point-of-care viral load allows testing, results feedback, and responsive care recommendations at the same visit
**Task Shifting**	Community health care workers delivering care because drugs are safe and monitoring is simple

## Here and Now: Eliminating Paediatric HIV

The most dramatic alteration in the HIV epidemic has been the marked reduction in HIV infection in children [2]. The Promise study, a recent randomised clinical trial conducted in Africa, shows that a globally applicable ART regimen is available that could reduce mother-to-child transmission of HIV during pregnancy or delivery to less than 1% [58]. A multicountry pre-exposure prophylaxis trial in infants found similar infection rates of 1.4% and 1.5% in the two arms at 50 weeks [59] and provides an alternative preventive option for women who are not ready for a lifetime commitment to ARV treatment as part of a multipronged prevention of mother-to-child transmission (PMTCT) programme [60].

These findings must be rapidly operationalised to curtail the quarter of a million new childhood infections that occur annually. Here, again, the vexing issue is access, highlighting the kind of health infrastructure investments required to identify and treat all pregnant women with HIV (Table 2). South Africa is the first country to report on population-level effectiveness of a national PMTCT programme [61] and has demonstrated a consistent trend toward lowering transmission among infants, with rates of 2.6% in 2012–2013 [61,62]. The effectiveness of PMTCT interventions in the field are influenced by access to prenatal care, retention in care, ARV adherence, adequate education, and follow-up of the mother and infant post-delivery (Table 2). To achieve adequate viral load suppression at the time of delivery, ARVs should be initiated early in pregnancy [12]. Even in South Africa, where over 85% of pregnant women attend at least four antenatal care visits, in 2014–2015 around half (54%) of pregnant women received their first antenatal care visit before 20 weeks of pregnancy [63].

**Table 2:** Potential demand-side and health system innovations to control paediatric HIV.

Innovation	Benefits
Scaling up community-based delivery platforms [64]	Access to universal health coverage
Training of community health workers [65]	Improve coverage along the continuum of care through early identification of pregnant women, encouragement of early antenatal booking, HIV testing, initiation of ARV treatment, and support for lifelong adherence to medication
Universal testing of infants [66]	Increasing access to the early identification of HIV infected infants and rapid initiation of ARV treatment [67]

Incident cases of HIV occurring during pregnancy and lactation increase the risk of mother-to-child transmission. Implementing repeat HIV testing in late pregnancy and during breastfeeding will be a further critical step required on the road to elimination [68]. Recommendations to repeat HIV testing around 32 weeks of pregnancy have been erratically implemented in South Africa, with only 22% of women in 2012–2013 reporting that they had their last HIV test at or beyond 32 weeks [62].

Hopefully, as more women are initiated on lifelong treatment, the continuum of care will improve so that prevention benefits are realised. One report from Malawi, however, acts as a cautionary tale: one in ten women were lost to follow-up six months after ART initiation (Table 3) [69]. Furthermore, solving the issues of health care stigmatization, access, status disclosure, and spousal involvement will also be needed to improve treatment initiation, adherence, and retention in care [70].

**Table 3:** Challenges in keeping HIV-positive pregnant women in care [69].

• Women initiated on lifelong ARVs during pregnancy were five times more likely than women who started ARVs in WHO stage 3/4 or with a CD4 cell count of 350 cells/ml or less to never return after their initial clinic visit.
• Women initiating lifelong ARVs while breastfeeding were twice as likely to miss their first follow-up visit.
• Loss to follow-up was highest in pregnant women who began lifelong ARVs at large clinics on the day they were diagnosed with HIV.

Because ART has successfully reduced MTCT and lowered morbidity and mortality in pregnant women, it has also led to growing numbers of HIV-exposed but uninfected children. Increased morbidity and mortality amongst HIV-exposed uninfected children has been described, including birth defects [71], small for gestational age (SGA) infants [72], growth [73] and neurodevelopmental delay [74], and reduced immunity to vaccine-preventable diseases [75,76]. There is an urgent need to better understand the health consequences of exposure to HIV and antiretroviral drugs on HIV-uninfected children and to improve monitoring and management of this growing subpopulation of children.

## HIV Cure

HIV infection results in latent infection of a select population of memory CD4+ T cells within days post-acquisition [77]. When the integrated HIV proviral DNA is transcriptionally silent, these resting cells are not affected by ARVs and are not recognised by the immune system. HIV can reactivate from these cells, however, and their presence constitutes a major barrier to eradicating infection [78,79]. Advances in understanding the basic biology of HIV have provided a framework to conceptualise novel approaches to curing the disease. These include genetic engineering technologies to either protect CD4+ T cells from future infection or deliver probes to inactivate integrated HIV DNA in the cell [80]. Use of genetically engineered T+ cells to provide long-term immune surveillance or to activate latently infected cells and destroy them and any associated HIV virions before they proliferate are being investigated [80]. Whether these strategies can either singly or in combination result in a true cure or what the field has termed a “functional” cure (that is, the ability to maintain viral suppression without ARVs with no risk of sexual or perinatal transmission) remains to be determined [80]. The benefits of such an approach on interrupting the spread of HIV are enormous and warrant a full-scale effort by the international scientific community.

## Conclusion

HIV brought an unprecedented shift in the global landscape of public health: it slashed life expectancy and wiped out a generation of economically active adults in their prime in sub-Saharan Africa; it reversed gains in under-five mortality and created a cohort of AIDS orphans; and it exposed the underbelly of poor health systems. It has also revealed the interrelatedness between social behaviour, stigmatization, cultural mores, religious beliefs, and human health. HIV has challenged all societies in our attempts to deal with its economic, societal, and medical aspects.

**Fig 2:**
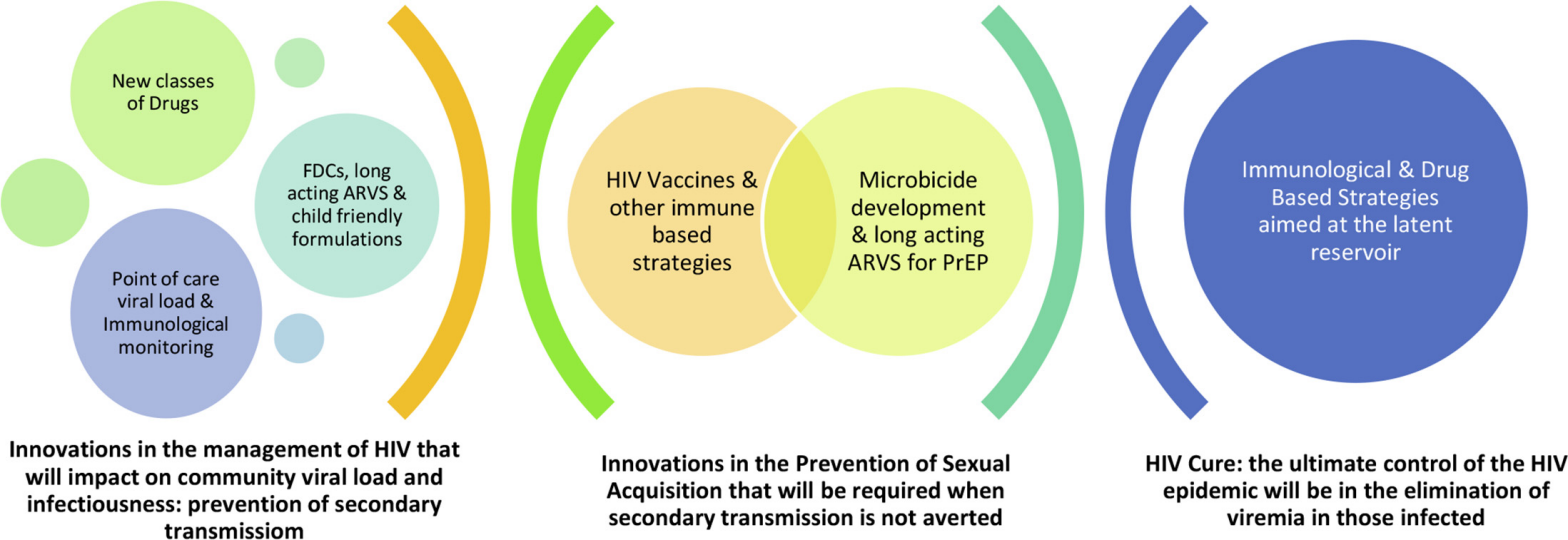
The spectrum of biomedical innovation required to end AIDS. Image credit: Glenda Gray.

Even with all the biomedical and behavioural tools available today, HIV continues to be a formidable pathogen, altering the health economics and public health strategies of most countries worldwide. Of the 35 million HIV-infected individuals worldwide in 2014, more than half did not know their HIV status, and over a third were not receiving ARVs [1] despite the availability of affordable point-of-care diagnostics and treatments. Adoption of “universal test and treat” approaches, extension of medical male circumcision programs, universal access to basic harm reduction services for people who inject drugs, and more widespread use of PrEP in targeted populations can do much to “bend the curve” and initiate a process to slow the rate of new HIV infections (Table 4). Such efforts are imperative on a global scale. However, it must also be recognised that true containment of the epidemic requires the development and widespread implementation of a scientific advance that has eluded us to date—a highly effective vaccine. There are potential synergies that will accrue from an integrated approach involving treatment, microbicides, and HIV vaccines (Fig 2). Various mathematical models agree that treatment roll-out on its own will decrease HIV incidence over time. However, when a 30% preventative HIV vaccine was introduced to a model with expanding treatment access in southern Africa, incidence was predicted to be 67% lower over time compared to a scenario with no vaccine introduction [35].

**Table 4:** Current biomedical interventions that are capable of bending the HIV epidemic curve.

**Intervention**	**R&D innovation**	**Source of financing**
1. Health system strengthenening	Smart clinic approaches; tools for procurement; health system processes development	Financial investments in health infrastructure, human resource development, health care worker training, continual monitoring and evaluation
2. Universal access to frequent HIV testing*	Over-the-counter HIV diagnostics, R&D into non-blood-based diagnostics, Point of care HIV diagnostics for infants, developing HIV counselling on mobile platforms.	Government & donor investment into biotechnology that simplifies point of care diagnostics whilst maintaining sensitivity and specificity, training of health care workers and community health workers on the mass rollout of HIV testing, values clarification for those carrying out HIV testing to reduce stigmatizing attitudes
3. Making ARV treatment available to all HIV-infected individuals, irrespective of CD4 count	R&D in fixed drug combinations, long-acting ARVs with high barriers to resistance and low side effects	Pharmaceutical investment with government subsidies, low cost of ARVs, task shifting to community health care workers
4. Medical male circumcision for neonates, adolescent boys, and adults*	Low-cost devices for male adults and neonates to allow mass medical circumcision without doctor supervision	Medical device investment, training health care workers in neonatal circumcision, task shifting.
5. Rolling out PrEP*	Continue R&D into long-acting ARV agents	Pharmaceutical investment with government subsidies
6. Universal PMTCT*	Safe fixed-dose combination (FDC) ARV, point of care virological and immunological monitoring tools	Government commitment to implementing HIV testing at family planning, improving access to safe contraception, allowing for safe termination of pregnancy, ARVs for life, task shifting.
7. Safe needle exchange*	Cheap disposable needles	Government support of clean needle exchange programmes

*Condom provision at every opportunity

## Abbreviations

ADCC: antibody-dependent cellular cytotoxicity
ART: antiretroviral therapy
ARV: antiretroviral
CMV: cytomegalovirus
FDC: fixed-dose combination
GMP: good manufacturing practice
IAVI: International AIDS Vaccine Initiative
MSM: men who have sex with men
MTCT: mother-to-child transmission
MVA: Modified Vaccinia Ankara
NIH: National Institutes of Health
NHP: nonhuman primate
NRTI: Nucleoside/Nucleotide Reverse Transcriptase Inhibitor
P5: Pox Protein Public Private Partnership
PMTCT: prevention of mother-to-child transmission
PrEP: pre-exposure prophylaxis
SGA: small for gestational age
SHIV: simian-human immunodeficiency virus
SIV: simian immunodeficiency virus
TAF: tenofovir alafenamide fumarate
TDF: tenofovir disoproxil fumarate
USMHRP: US Military HIV Research Program
